# Forty-Fourth Annual Meeting of the Mississippi Valley Association of Dental Surgeons

**Published:** 1888-05

**Authors:** A. G. Rose


### PROCEEDINGS.

### Forty-Fourth Annual Meeting of the Mississippi Valley Association of Dental Surgeons.

The forty-fourth annual meeting of this body was called to order promptly at 10 o'clock A. M., March 7th, 1888, by First Vice-President Wm. N. Morrison, President Harlan being absent, the following communication from him was read:

ROME, ITALY, Feb. 22d, 1888.

*To the Mississippi Valley Association of Dental Surgeons:*

Finding the time approaching when your association will meet again in annual convention, and your president being in foreign lands he thought you would be glad to hear from him, and consequently I write to say that he is in very good health, thanks to few weeks rest and recreation from ordinary occupation of practice, lectures, and editing a dental journal. I sincerely hope that the members of the oldest dental association in the world are in good health and prospering. Now that I cannot b[illegible] with you and preside over your deliberations, I wish to write [illegible] line or two about things I have heard since traveling on this [illegible] of the water. Almost every genuine American dentist or[illegible]

continent and in Great Britain also is complaining of some American Dental colleges graduating foreigners on a few weeks attendance at our schools, and with little or no familiarity with the English language. It is claimed that in a short time, (six or eight weeks) some of them return with a D.D.S. and an M.D. degree also, from good schools. I have heard it stated that in some cases the foreign student goes over to America and matriculates, stays a few weeks, returns home and the next year he receives his degree. This is done (so it is claimed) in order to evade the requirements of the National Association of Dental Faculties. In regard to this matter I desire to say that the Mississippi Valley Association of Dental Surgeons ought to adopt resolutions of a very strong character declining to admit to membership a graduate of any college guilty of such reprehensible practices. The American D.D.S. is now looked upon with some shrugging shoulders by many foreigners and if we at home can begin the needed reform it is our duty to do so, and not wait until compelled by the enactment of law in foreign lands to raise the standard so as to compare to them.

It is not the genuine American D.D.S. who brings discredit upon the title, but the foreigner who calls himself American dentist, and who in many cases has a genuine American degree that bedraggles the title and the name in the dust.

Hoping that this will receive and merit your consideration and wishing you a pleasant and profitable meeting, I am very sincerely yours,

A. W. Harlan,
President M. V. D. Ass'n.

---

A motion was carried instructing the Secretary to make a list of the names of those present. Dr. J. Taft moved that a committee consisting of the Secretary, Treasurer, and one other be appointed by the Chair and empowered to revise the existing constitution, or if need be, present a new one, also to make a [illegible]mple[illegible] list of the present membership, with the understanding [illegible]at any member in arrears of dues for two years be stricken from [illegible] roll. Carried.

[illegible]he Chair appointed Dr. Will Taft on the committee with [illegible]tary and Treasurer.

The minutes of last year's meeting were read and adopted. First Vice-President Morrison then read an address published in the April number of the REGISTER.

Dr. C. M. Wright, Chairman of the Executive Committee reported the following programme:

"*The Dental Pulp*,"
JUNIUS E. CRAVENS, D. D. S., Indianapolis, Ind.
J. H. CALLAHAN, D. D. S., Hillsboro, Ohio.

"*Immediate Root Filling*,"
H. A. SMITH, D. D. S., Cincinnati, Ohio.

"*Implantation*," with Clinical Demonstrations,
M. H. FLETCHER, D. D. S., M.D., Cincinnati, Ohio.

"*Combination Fillings*,"
H. J. McKELLOPS, D. D. S., St. Louis, Mo.

*Constitutional Aspects of "Pyorrhea Alveolaris*,"
J. H. CALLAHAN, D. D. S., Hillsboro, Ohio.

"*Incidents of Office Practice*,"
Several voluntary Papers from distinguished writers promised.

*Prize Essay Subject:*

"The Causes of Deposits on the Teeth, and Methods of Removing the Same."

*Voluntary Papers:*

A Paper on "GANGRENOUS TOOTH-PULPS, AS CENTERS OF INFECTION," is promised from *Dr. W. D. Miller*, of Berlin,; also a Paper from *Dr. N. W. Williams*, of Geneva, Switzerland, and one from *Dr. L. Custer*, of Dayton, Ohio.

In addition, there were other papers received too late to be listed.

Dr. Rose, of the Publication Committee, read a letter from the editor of the British Journal of Dental Science, asking that full reports of the meeting, and papers read be transmitted to him for publication in said journal. It was referred to Publication Committee, with power to act.

The Membership Committee presented the following names of new members, all of whom were elected by Secretary's ballot:

Wm. Conrad, St. Louis; R. H. Updegraff, Hutchinson, Kansas; E. P. Binford, Cincinnati; Wm. Ames, Chicago; W. J. Zinn, Williamstown, Kentucky; S. J. Hayes, Pittsburg, Pennsylvania; B. Stevens, Hannibal, Missouri; H. J. Matlack, Covington, Kentucky; H. T. Smith, Jos. Silcott, W. M. Williams, Mrs. W. B. Swift and Mrs. Jessie Dillon, all of Cincinnati.

Committee on ethics had no report.

Treasurer Hunter reported a balance of $68.00.

Chair appointed Drs. H. L. Moore and J. R. Callahan a Committee to audit the accounts.

On motion of Dr. J. Taft, the hours of the session were fixed at 9 to 12 A. M., and 2 to 5:30 P. M.

On motion of Dr. McKellops, 11 o'clock Thursday was set apart for the exhibition of appliances, also that 4 o'clock Wednesday be set apart to hear some letters from the counsel engaged in the suit now pending in the Supreme Court relative to the Richmond Crown and Low Crown Bridgework patents and the tariff on dental goods and appliances.

On motion of Dr. Fletcher. Dr. Sage, Betty, and Wright were appointed a committee to nominate delegates to the American Medical Association in accordance with a unanimous resolution passed by that body; one delegate for each ten of the membership.

Dr. E. G. Betty presented the following memorial on the death of Dr. Walter A. Dunn:

WHEREAS, This Society learns with deep regret of the death of Dr. Walter A. Dun, a physician who recognized the importance of Dentistry as a branch of the Healing Art, and who used his influence to further its standing as a science, and, whereas he having participated in the deliberations of this Society at its last meeting be it

*Resolved*, That the Association extend to his family its sympathy in their bereavement, and further

*Resolved*, That a suitable appropriation be made towards the fund for the endowment of a memorial bed in the Child's Hospital at Mt. Auburn, in which he was greatly interested, and be it further

*Resolved*, That, this resolution be incorporated in the minutes of the Association and a copy transmitted to the family of the deceased and publication be made in the Journals.

The Auditing Committee reported the Treasurer's books correct. Discharged.

FIRST DAY AFTERNOON SESSION.

Meeting called to order by Vice-President Morrison at 2 P. M. Reading of minutes dispensed with. Dr. W. S. Howe, of Philadelphia, read a paper on "Professional Patents"; Dr. J. R. Callahan read one on "The Dental Pulp"; Dr. C. M. Wright read a paper written by Dr. Wm. H. Pease, of Dayton, Ohio, on the same subject; he also read one from Dr. Miller, of Berlin, upon "Gangrenous Tooth-Pulps as Centers of Infection"; Dr. H. A. Smith read a paper upon "Immediate Root Filling."

The papers were discussed by Drs. Conrad, Berry, Watt, Leslie, Callahan, Moore, Betty, Sage, and J. Taft.

Dr. E. G. Betty to the letter from the President of the Association, Dr. A. W. Harlan, calling special attention to the fact that some Dental Colleges of the United States were issuing diplomas conferring the degree of Doctor of Dental Surgery upon an attendance at said colleges of not more than three weeks and that such a condition was hurtful to our regular practitioners abroad, and calling for action in the matter. After some discussion, Dr. J. R. Callahan withdrew a motion he had made in regard to the matter with the understanding that he would next morning present it in due form in writing. The hour of special business having arrived, Dr. H. J. McKellops spoke at some length on the position of the dental profession with the relation to the suit now pending in the Supreme Court of the United States—Crown and Bridgework Patent cases. Several letters were read stating the case as it now exists and appeals for the sinews of war, *i. e.*, money, from Solomon J. Gordon, counsel for the dental profession. The subject was freely discussed. Dr. E. G. Betty offered the following:

*Resolved;* That this Society pledges itself to appropriate $100 toward the fund to defend the interests of the dental profession in the Crown and Bridge patents, $50 of which can be paid at once by the Treasurer. Carried.

On motion, Drs. Wright and Fletcher were appointed a committee to take charge of said fund and the subscriptions made for the same object. Adjourned.

SECOND DAY MORNING SESSION.

The society met according to adjournment with Vice President Morrison in the chair; the minutes of the previous session were read and adopted.

Dr. E. G. Betty offered the following resolution:

*Resolved*, That this Association officially condemns the action of many American Dental Colleges in issuing diplomas to foreigners or others who have not attended the full courses as prescribed and provided for by the National Association of Dental College Faculties, inasmuch as this has brought the genuine American dentists abroad into disrepute in foreign countries, and be it further

*Resolved*, That this society appoint a committee of three to confer with the National Association of Dental Examiners to single out such colleges and debar their graduates from practicing in any of the United States represented in said Association, and be it further

*Resolved*, That the members of this society withhold their patronage and influence from any such colleges.

After much discussion, the resolution was, on motion of Dr. F. A. Hunter, laid upon the table.

Dr. C. C. Carroll, of Meadville, Pa., gave an exhibition of his method of casting aluminum plates. Dr. S. J. Hayes, of Pittsburgh, Pa., exhibited his appliance for producing anæthesia. Dr. H. J. McKellops exhibited Dr. L. D. Shepard's impression cups and regulating device, Dr. W. E. Ellis' separators, Dr. Miller's disc holder and Williams' crystalloid gold for use by mallet or hand pressure. Upon request Dr. McKellops spoke of the First District Dental Society's clinics recently held in New York City. Dr. W. S. Howe exhibited Perry's separators, Howe's clamp and mode of mounting Logan tooth crowns. Dr. Morrison exhibited a preparation of mineral wool for use in connection with oxychloride of zinc in pulp canals. Dr. M. H. Fletcher read an interesting paper upon "Implantation" and under very unfavorable circumstances successfully performed the operation in the mouth of a young man who said the pain was only moderate, a ten per cent. solution of cocaine being used as an obtundent; the usual procedure in the operation was followed. Adjourned.

### SECOND DAY AFTERNOON SESSION.

The Association met with President and Secretary at their posts; minutes read and approved. Dr. Sage reported the following delegates to the American Medical Association: H. J. McKellops, J. E. Cravens, W. B. Ames, J. R. Callahan, Geo. Watt, H. A. Smith, H. L. Moore, E. G. Betty, J. Stoddard Driggs, and A. O. Rawls.

On motion of Dr. A. G. Rose, the report of the committee was adopted. On motion of Dr. Watt the delegates appointed were empowered to elect other members to fill vacancies, if any occur in the delegation at the time of meeting. Carried.

Dr. J. Taft moved that Drs. Sage, Betty and Wright be requested to nominate delegates to represent this body at the meeting of the American Dental Association to be held in Louisville, Ky., in August. Carried.

Dr. J. E. Cravens, of Indianapolis, read a synopsis of a paper he was prevented from presenting to the Society in complete form upon the "Dental Pulp."

A discussion on the first and second topics of the programme was announced in order by the Chairman, and the following gentlemen took part: Drs. Wm. H. Atkinson, Conrad, Jay, Sage, Cravens, Taft, Watt, Smith and Fletcher.

On motion topics passed and the third subject on the programme considered.

Dr. Atkinson, by request, addressed the Association upon the subject of Implantation.

The advisability of holding a night session was discussed and a motion to hold such session was lost.

On motion eleven o'clock Friday morning was made a special hour of business for the election of officers.

Adjourned.

THIRD DAY—MORNING SESSION.

Dr. Morrison in the Chair ; Dr. A. G. Rose, Secretary.

On motion, minutes was adopted as read. Committee on Revision of Constitution were instructed to frame a by-law or article prohibiting gentlemen joining this Society for the purpose of advertising their special wares or patents and to protect the members from imposition.

Dr. Wright, of the Executive Committee, read a communication from Dr. N. W. Williams, of Geneva, Switzerland. Dr. Taft moved that the Corresponding Secretary be instructed to forward a suitable letter of thanks. Carried.

Dr. L. E. Custer, of Dayton, O., read a paper on "Intermittent Pressure ; Its Relation to Orthodontia."

The subject of combination fillings coming up, the Chairman of the Executive Committee read a selection from the pen of Dr. W. D. Miller, of Berlin, on the use of tin and gold combined.

Dr. J. W. Jay, of Richmond, Ind., read a paper under the heading of "Incidents of Office Practice," citing cases of resuscitation from anæsthesia.

Dr. E. G. Betty offered the following resolution which was unanimously adopted : *Resolved*, That this Association desires to put itself on record as protesting against the action of those colleges of the United States which issue diplomas to foreigners and others without having attended a full course of lectures, as provided for in the curriculum adopted by the National Association of Dental College Faculties, inasmuch as the procedure has brought into disrepute the genuine American dentists abroad, and be it further

*Resolved*, That the Association appoint a committee of three to bring the matter before the National Association of Examiners for their action." The Chair appointed Dr. Betty, Rose and Wright as said committee.

Dr. H. C. Matlack, of Covington, Ky., was elected to membership by cast of Secretary's ballot.

The Association proceeded to the election of officers of the Mississippi Valley Dental Association for the coming year, with the following result :

President, Dr. H. L. Moore.
First Vice-President, Dr. J. R. Callahan.
Second Vice-President, Dr. M. H. Fletcher.
Corresponding Secretary, Dr. F. W. Sage.
Recording Secretary, Dr. A. G. Rose.
Treasurer, Dr. F. A. Hunter.

The following committees were appointed:—Executive, Drs. Betty, Heise, and Conrad; On Publication, Drs. Custer, Morrison, and Williams; On Membership, Drs. Taylor and Taft; On Ethics, Drs. Craven, J. Taylor, and Binford.

On motion it was decided that notification shall be made to members appointed as delegates to the Dental Section of the American Medical Association, which meets in this city in May, and also to the American Dental Association which meets in Louisville, Ky., during the first week in August. Bills for printing and postage were ordered paid on motion to that effect.

On motion the society adjourned to meet the first Wednesday in March, 1889.

A. G. Rose,
Recording Secretary,
per E. G. Betty.

